# Impaired activation of succinate-induced type 2 immunity and secretory cell production in the small intestines of *Ptk6−/−* male mice

**DOI:** 10.1038/s41419-024-07149-9

**Published:** 2024-10-26

**Authors:** Katarina Vlajic, Wenjun Bie, Milica B. Gilic, Angela L. Tyner

**Affiliations:** 1 Department of Biochemistry and Molecular Genetics, University of Illinois College of Medicine, University of Illinois at Chicago, Chicago, IL 60607 USA; 2 Present Address: University of Washington, Seattle, WA USA; 3 Present Address: St Jude Children’s Hospital, Memphis, TN USA

**Keywords:** Biochemistry, Cell signalling, Innate immune cells

## Abstract

Protein tyrosine kinase 6 (PTK6) is an intracellular tyrosine kinase that is distantly related to the SRC family of tyrosine kinases. It is expressed in epithelial linings and regulates regeneration and repair of the intestinal epithelium. Analysis of publicly available datasets showed *Ptk6* is upregulated in tuft cells upon activation of type 2 immunity. We found that disruption of *Ptk6* influences gene expression involved in intestinal immune responses. Administration of succinate, which mimics infection and activates tuft cells, revealed PTK6-dependent activation of innate immune responses in male but not female mice. In contrast to all wild type and *Ptk6*−/− female mice, *Ptk6*−/− male mice do not activate innate immunity or upregulate differentiation of the tuft and goblet secretory cell lineages following succinate treatment. Mechanistically, we found that PTK6 regulates *Il25* and *Irag2*, genes that are required for tuft cell effector functions and activation of type 2 innate immunity, in organoids derived from intestines of male but not female mice. In patients with Crohn’s disease, PTK6 is upregulated in tuft cells in noninflamed regions of intestine. These data highlight roles for PTK6 in contributing to sex differences in intestinal innate immunity and provide new insights into the regulation of IL-25.

## Introduction

The rapidly regenerating intestinal epithelium contains anchored stem cells that give rise to absorptive cells and different secretory cells, including enteroendocrine, goblet, Paneth, and tuft cells. Mucus-secreting goblet cells and anti-microbial peptide-secreting Paneth cells are well-recognized regulators of host-microbe interactions [1, 2]. More recently, tuft cells were identified as activators of innate immunity and part of the surveillance system for extracellular pathogens such as helminths, protists, and bacteria [3–6]. Emerging studies also suggest roles for tuft cells in repair processes, such as the attenuation of chronic inflammation [7].

Tuft cells are rare epithelial cells in the intestine under normal conditions. They specifically express interleukin (IL)-25, a cytokine that is necessary for the activation of innate immunity [5]. Tuft cells express G protein-coupled receptors such as taste receptors [8, 9], the succinate receptor 1 (SUCNR1) [10–12], or the vomeronasal receptor Vmn2r26 [6], which detect pathogen metabolites in their environment. In mice, administration of the metabolite succinate in drinking water is sufficient to induce an innate immune response in the intestine [10–12]. Following activation of these receptors, tuft cells secrete IL-25 to alarm immune cells in the lamina propria that pathogens are present. IL-25 signals to innate lymphoid cells type 2 (ILC2s) to produce IL-13 and activate the immune cascade [5]. IL-13 drives the type 2 immune response through the recruitment of other immune cells and upregulation of Gasdermin C proteins (GSDMCs), which have roles in pyroptosis, caspase-1-dependent programmed cell death associated with inflammation [6, 13]. In addition, IL-13 promotes secretory cell differentiation, including differentiation of tuft and goblet cells that increase in number [3–5]. Increases in mucus production, fluid secretion, and muscle contraction generate a “weep and sweep” response to eliminate infections [14, 15].

Protein tyrosine kinase 6 (PTK6, also known as BRK or Sik) is an intracellular non-receptor tyrosine kinase distantly related to the SRC family, which belongs to the PTK6 family of protein kinases together with FRK and SRMS (reviewed in [16]). PTK6 is expressed in regenerating epithelial linings of the skin and gastrointestinal tract [17–19] and has been shown to regulate intestinal growth and differentiation [18, 20]. PTK6 regulates the WNT/β-catenin signaling pathway [21], as well as signaling downstream of several growth factor receptors, including EGFR, IGF1R, and MET (reviewed in [16]). PTK6 expression can be induced by different stress signals, including DNA damage, from γ-irradiation [22, 23], ultraviolet radiation [24], azoxymethane [25], to hypoxia [26]. Damaging signals lead to the upregulation of PTK6 in the progenitor cell/stem cell compartment in the intestine, to promote regeneration and repair of intestinal epithelium [22, 25].

New bulk RNA sequencing (RNA-seq) data revealed differences in mucosal innate immune response gene expression in mice with germline disruption of *Ptk6*. In publicly available datasets [27, 28], we detected increased *Ptk6* expression in the intestine following infections with the nematode parasite *Heligmosomoides bakeri* (Hb) or *Salmonella*, different activators of type 2 immunity. Here we demonstrate that PTK6 is essential for activation of type 2 immunity in male but not female mice. These data reveal important mechanistic differences in activation of type 2 immunity between sexes, which may have an impact on sexual disparities in disease pathogenesis.

## Results

### PTK6 regulates sex-specific gene expression in the intestine

**Fig. 1:**
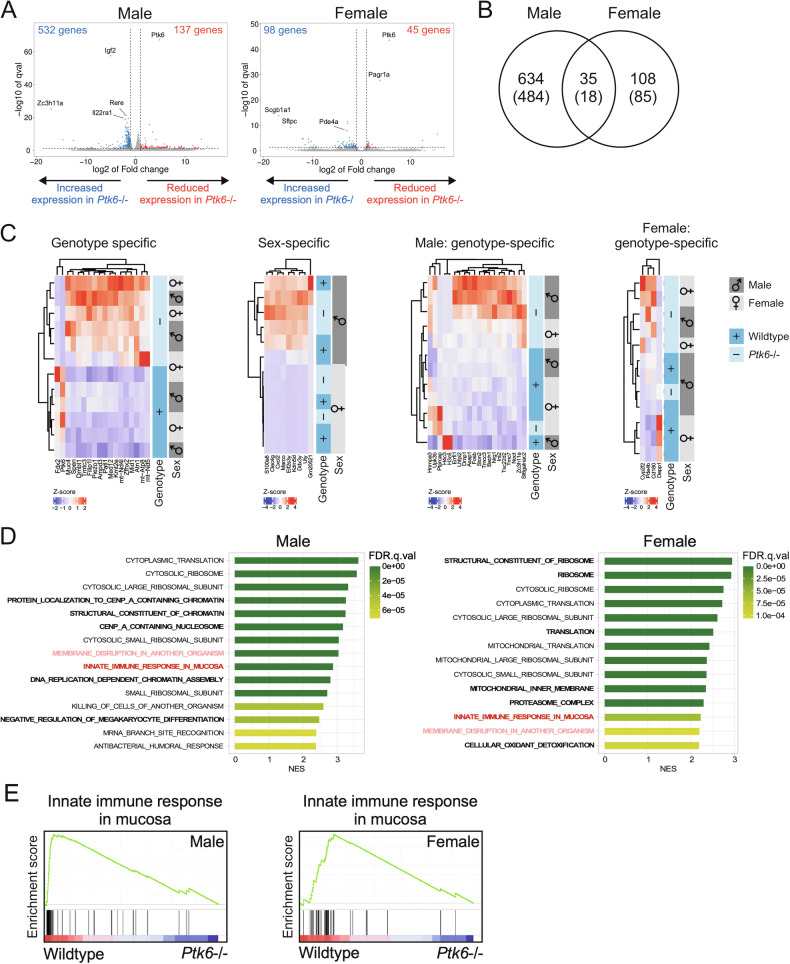
PTK6 regulates sex-specific gene expression and innate immunity genes in the intestine. **A** Analysis of jejunal RNA expression in male and female wild type and *Ptk6*−/− mice suggests unique roles for PTK6 in males and females. The volcano plots of differentially expressed genes (DEGs) show that male mice have more DEGs than female mice, with more genes changing in expression in male mice upon *Ptk6* loss. (N = 3 per group). **B** Male and female wild type and *Ptk6*−/− mice share only 35 genes among significantly DEGs. A Venn diagram shows number of male DEGs only, female DEGs only and mutual DEGs in the overlap. The number of all genes is shown on top, while protein coding genes are shown below in parenthesis. **C** Heatmaps show expression of significant DEGs between genotypes, sexes, as well as significantly different genes in males and females that are genotype specific. **D** Gene set enrichment analysis of genes expressed in male and female wild type and *Ptk6−/−* mice. Innate immune response in mucosa (bold red) is one of the top different gene sets in both male and female mice, based on normalized enrichment score (NES). Another top gene pathway is “Membrane disruption in another organism” (bold light red), which shares genes with “Innate immune response in mucosa”. Male mice have some specific gene sets that do not display significantly different gene expression in females, and vice versa (bold black). The top 15 gene sets are shown. NES represents normalized enrichment score to account for the gene set size. **E** The enrichment plots of the innate immune response in mucosa gene set from GSEA for male and female mice. The leading-edge subset shows a cluster of genes that are responsible for high enrichment scores (ES) in wild-type mice. For male mice: ES = 0.74977636, NES = 2.876303, NOM *p*-value < 0.0001, FDR *q*-value < 0.0001. For female mice: ES = 0.7265350, NES = 2.2186077, NOM *p*-value < 0.0001, FDR *q*-value < 0.0001.

Previous in vivo studies of PTK6 in the intestine utilized only male mice [18, 22, 25]. To determine if there are sex-specific differences in PTK6 functions, we performed bulk RNA-seq using total RNA from distal jejunums of male and female wild type and *Ptk6*−/− mice. Disruption of *Ptk6* led to more significant changes in gene expression in males than females. When compared with wild-type controls, increased expression of 532 genes was detected in male *Ptk6*−/− mice, compared with increased expression of only 98 genes in *Ptk6*−/− female mice (Fig. 1A). Similarly, loss of *Ptk6* led to decreased expression of 137 genes in males and 45 genes in females when compared with controls. Only 35 differentially expressed genes (DEG), of which 18 are protein coding, were shared between males and females (Fig. 1B, Table S1). One of the most significantly upregulated genes in *Ptk6−/−* male mice is *Il22ra1*, which is expressed in all cell types, but is required for IL-22-induced differentiation of Paneth cells [29], as well as a specific subtype of goblet cells [30]. Heatmaps show the expression of top significant protein-coding DEGs between genotypes, and sexes, as well as significantly different expressed genes in male and female tissues dependent on genotype (Fig. 1C).

To understand the differences between wildtype and *Ptk6*−/− mice, we used gene set enrichment analysis (GSEA). Transcriptome analysis identified the “innate immune response in mucosa” as one of the significantly different biological pathways between wild type and *Ptk6*−/− mice of both sexes (Fig. 1D, Table S1). Annotated genes included mostly Paneth cell genes (*Defa*) and some type 2 immunity genes (*Il4*), and the majority of these, including *Defa35*, *Defa27* and *Defa34*, were among the leading-edge genes in both male and female mice (Fig. 1E). Even though tuft and goblet cell marker genes were not in this gene set, there is a crosstalk between tuft and Paneth cells [31, 32], as well as tuft and goblet cells [5]. This suggested that PTK6 could have a role in the regulation of intestinal innate mucosal immunity responsible for defense against different pathogens, including bacteria, protists, helminths, fungi, and some viruses [4, 6, 33, 34].

### *Ptk6* is upregulated in tuft cells by pathogens that activate intestinal type 2 immunity

**Fig. 2:**
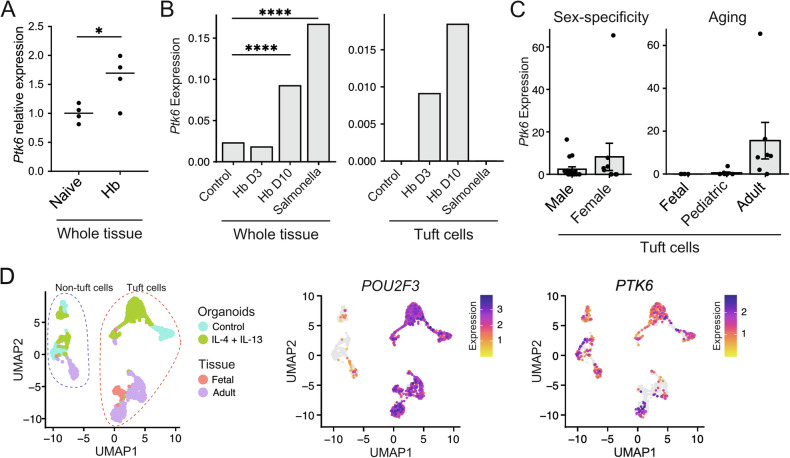
*Ptk6* is upregulated in tuft cells upon activation of type 2 immunity. **A** *Ptk6* mRNA expression increases in duodenum 24 h after infection with the helminth *Heligmosomoides bakeri* (Hb), shown as the change in relative expression from bulk-RNA-seq data [28]. **B** *Ptk6* expression increases by 10 days (D10) in whole intestinal tissue from mice infected with Hb or by 2 days following Salmonella infection [27]. Increased *Ptk6* expression was detected specifically in tuft cells in scRNA-seq data post Hb infection. *Ptk6* expression is shown as average expression from all cell types per condition (left) or for tuft cells only per condition (right). Significant upregulation of *Ptk6* at: day 10 upon *H. bakeri* infection p. adj = 2.120863e-15; day 2 upon Salmonella infection p.adj = 1.468545e-42. **C** In the human gastrointestinal tract, *PTK6* mRNA expression is upregulated with age in tuft cells, while it appears similar between sexes [35]. **D** *PTK6* expression is upregulated in tuft cells upon IL-13/IL-4 treatment of human intestinal organoids [36]. UMAP shows clustering of tuft and non-tuft cells, and their origins. *POU2F3* is shown as a marker for tuft cells. *PTK6* is predominantly found in organoid tuft cells, and to a lesser extent in tissue-derived tuft cells.

We examined publicly available datasets from different intestinal infection models for *Ptk6* expression. Analysis of RNA-seq data from mice infected with *Heligmosomoides bakeri* (Hb), showed increased expression of *Ptk6* at 24 h after infection (Fig. 2A) [28]. In a single cell (sc) RNA-seq study [27], we found *Ptk6* is significantly upregulated in small intestinal tissue at 10 days post-Hb infection (Hb D10) and 2 days after *Salmonella* infection (Fig. 2B). At the single-cell level, *Ptk6* expression exhibits an upward trend in tuft cells after Hb infection but was undetectable in tuft cells in uninfected controls and in *Salmonella*-infected intestines (Fig. 2B).

Exploring data from Elmentaite and colleagues, we detected *PTK6* expression in tuft cells in both male and female samples [35]. Interestingly, the majority of PTK6 expression was present in adult tuft cells (Fig. 2C). Upregulation of *PTK6* in human tuft cells upon activation of type 2 immunity is confirmed in single-cell RNA-seq data from organoids treated with IL-13 [36] (Fig. 2D). *PTK6* expression is particularly high in the population that is upregulated upon IL-4/IL-13 treatment. This suggested that PTK6 may play roles in regulating tuft cell differentiation and function.

### Male mice lacking PTK6 fail to upregulate differentiation of secretory cell lineages after succinate treatment

**Fig. 3:**
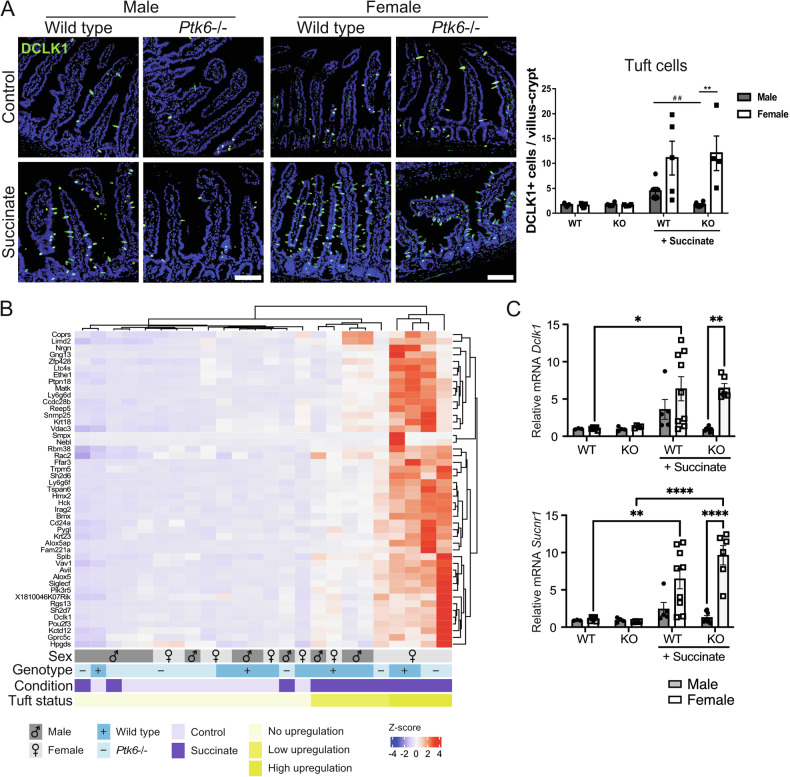
Tuft cell expansion is impaired in *Ptk6*−/− male mice after activation of type 2 immunity with succinate. **A** Induction of tuft cells is impaired in male *Ptk6*−/− (KO) mice following succinate treatment. Females exhibit an upward trend for stronger induction of tuft cells than males. Representative images of anti-DCLK1 staining in tuft cells of jejunal tissues from male and female wild type and *Ptk6*−/− mice. Scale bar, 50 *μ*m. Tuft cell numbers were determined as the total number of DCLK1+ cells per villus-crypt unit. **B** Expression of tuft cell marker genes identified by Haber et al. [27] was analyzed in jejunal RNA-seq data from male and female wild type and *Ptk6*−/− mice. Female wild type and *Ptk6*−/− mice exhibit strong upregulation of tuft cell marker genes, while male *Ptk6*−/− mice have impaired upregulation of these genes following succinate treatment. **C** Relative mRNA expression of tuft cell markers *Dclk1* and *Sucnr1*. qRT-PCR was performed with jejunal RNAs from wild type and *Ptk6*−/− male and female mice. Gene expression is normalized against *Rps17* and compared with wild type control mice. For all experiments: RNA-seq *n* = 3; quantification and qRT-PCR *n* = 3 to 9 mice. Graphs depict mean ± SEM. Statistics: Two-way ANOVA, adjusted for multiple comparisons using Tukey test. *p-value < 0.05, **^,##^*p*-value < 0.01, ****p-value < 0.0001. # is used for comparison within the same sex when shown on the same graph with all comparisons.

We used the succinate-based system to explore roles of PTK6 in type 2 immunity and tuft cell differentiation [10–12]. Wild type and *Ptk6*−/− mice were treated with succinate for 7 days and sections of jejunum were stained for expression of the tuft cell marker DCLK1, revealing sex-specific differences in induction of tuft cells. Male *Ptk6*−/− mice do not exhibit an increase in tuft cells following succinate treatment (Fig. 3A), while both female wild type and *Ptk6*−/− mice have more tuft cells, than male wild type mice (Fig. 3A). Likewise, tuft cell marker gene expression [27] is significantly upregulated in female jejunums following succinate treatment (Fig. 3B, Table S2). *Ptk6*−/− male mice treated with succinate express tuft cell marker genes similar to untreated males and females of both genotypes (Fig. 3B). We confirmed differences in expression of the tuft cell markers *Dclk1* and *Sucnr1* by qRT-PCR (Fig. 3C). Significantly upregulated genes in male and female wild type and *Ptk6*−/− female mice upon succinate treatment are predominantly part of the type 2 tuft cell gene signature [27] (Fig. S1A, B).

**Fig. 4:**
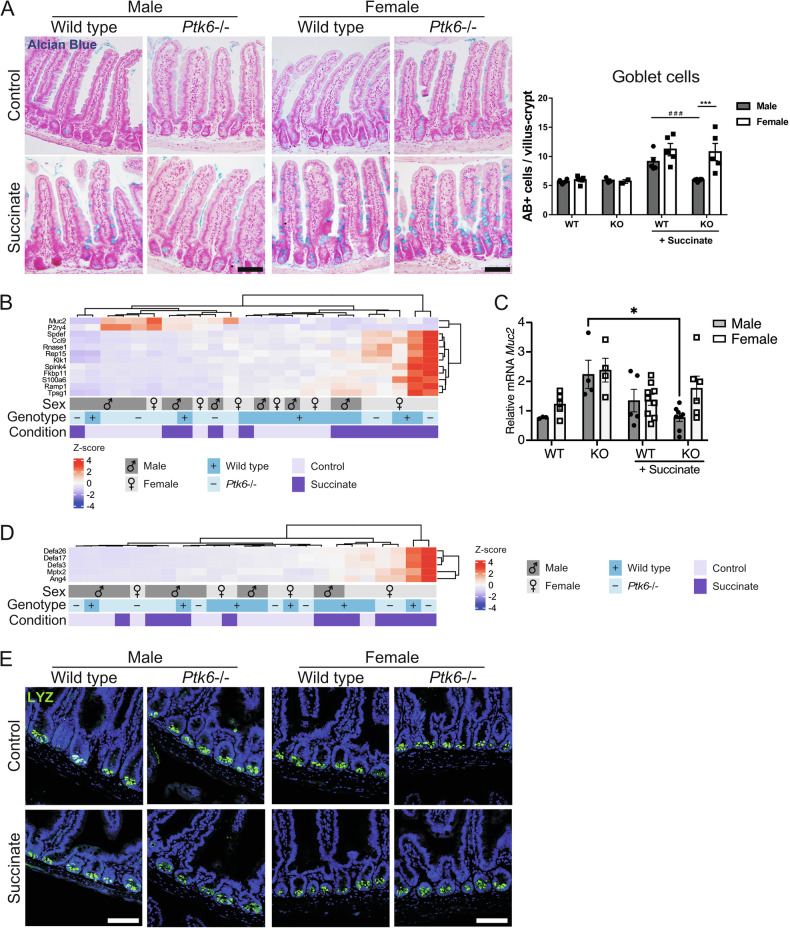
Male *Ptk6*−/− mice have impaired upregulation of goblet cells, as well as goblet and Paneth cell genes upon succinate treatment. **A** Expansion of goblet cells is impaired in male *Ptk6*−/− mice upon treatment with succinate. Representative images of Alcian Blue (AB) stained jejunum from male and female wild type and *Ptk6*−/− mice. Scale bar, 50 *μ*m. Quantification of goblet cells is shown as total number of AB+ cells per villus-crypt unit. ***^,###^ p-value <0.001. **B** Heatmap of significantly expressed goblet cell genes in RNA-seq data. *Ptk6*−/− control mice express higher levels of *Muc2* and *P2ry4*, goblet cell genes, compared with succinate treated mice. **C** qPCR was used to validate *Muc2* expression; relative *Muc2* mRNA expression is normalized against *Rps17*. *p-value < 0.5. **D** Expression of Paneth cell markers, identified by Haber et al. [27]. **E** There is no change in Paneth cell numbers upon succinate treatment for 7 days. Jejunums were stained for expression of lysozyme (LYZ), a Paneth cell marker. Scale bar, 50 μm.

Since goblet cell expansion is characteristic for activation of type 2 immunity [3–5], we examined goblet cell differentiation following succinate treatment of wild type and *Ptk6*−/− mice. In human tissues, *PTK6* is found in both male and female goblet cells and is upregulated with aging (Fig. S1C), consistent with upregulation of secretory cell lineages with aging [37]. In jejunal sections stained with Alcian Blue to detect mucins, we observed sex differences in goblet cell numbers upon activation of type 2 immunity with succinate (Fig. 4A). The numbers of goblet cells in wild type and *Ptk6*−/− female mice show a positive upward trend, in comparison with wild type males. However, male *Ptk6*−/− mice do not exhibit any expansion of goblet cells (Fig. 4A). Several goblet cell marker genes are upregulated like tuft cell-related genes after the succinate treatment, particularly in female mice of both genotypes (Fig. 4B). In contrast, *Muc2* (mucin 2) and *P2ry4* (pyrimidinergic receptor P2Y4), expressed by goblet cells, are more highly expressed in untreated male and female *Ptk6*−/− controls, and their expression is reduced upon succinate treatment (Fig. 4B). Higher *Muc*2 mRNA expression was validated by qPCR (Fig. 4C). *Muc2* expression and goblet cells were upregulated in *Ptk6−/−Frk−/−Srms−/−* mice [38], suggesting that PTK6 may be a suppressor of *Muc2* production, although loss of *Ptk6* alone was not sufficient to upregulate basal goblet cell numbers.

Paneth cell marker genes are upregulated in wild type and *Ptk6*−/− female mice (Fig. 4D), but staining of jejunal sections with antibodies against lysozyme, a Paneth cell marker, did not show any changes in Paneth cell numbers following succinate administration (Fig. 4E), as has been previously described [5]. This may be due to the long life of Paneth cells, which have a turnover time of up to 2 months [39]. Additionally, we did not observe differences in cell proliferation, identified by Ki67 staining (Fig. S2), and we did not detect any significant changes in the expression of proliferation markers in RNA-seq analysis, including *Mki67*, *Pcna*, and *Mcm2*.

### Type 2 immunity genes are significantly upregulated in female mice following succinate treatment

**Fig. 5:**
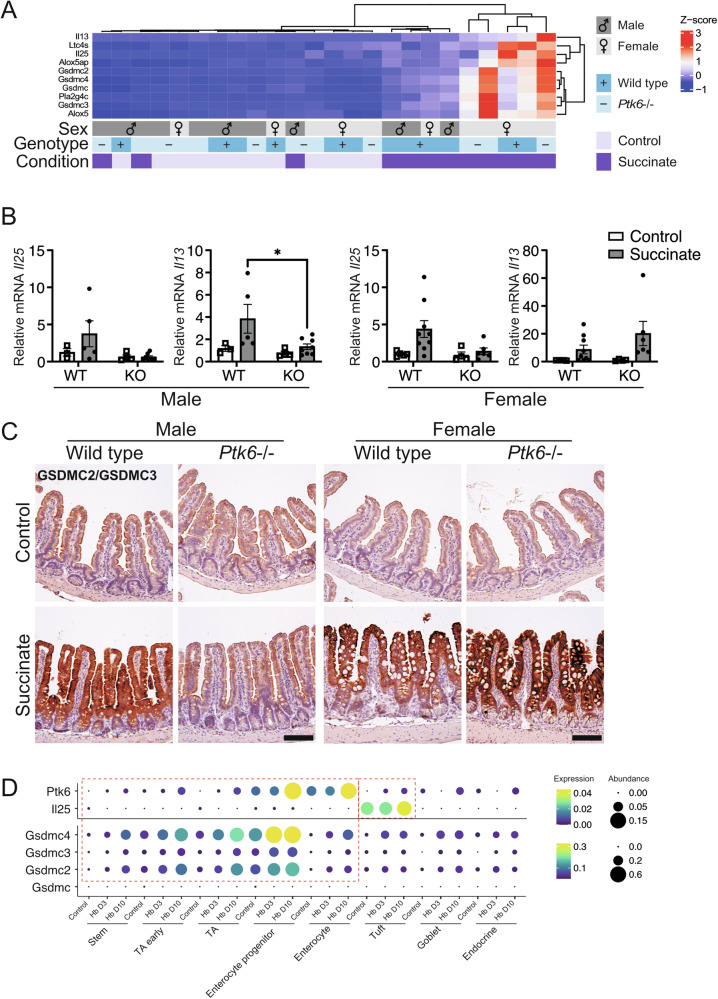
Tuft cell effector functions are impaired in male *Ptk6*−/− mice. **A** A heatmap shows a significant increase in expression of tuft cell effector and type 2 immunity genes in succinate-treated female mice, and impaired upregulation in male succinate-treated *Ptk6*−/− mice. Gasdermin C genes (*Gsdmc1-4*) involved in type 2 immunity related pyroptosis, and *Pla2g4c* are some of the most significantly upregulated genes in the dataset. **B** Examination of gene expression using qRT-PCR shows impaired induction of type 2 immunity cytokine genes in male *Ptk6*−/− mice. Relative expression of *Il25* from tuft cells, and *Il13*, an ILC2-produced cytokine needed for differentiation and expansion of tuft cells, in male and female mice is shown. Gene expression is normalized against *Rps17*. Graphs depict mean ± SEM. Statistics: Two-way ANOVA, adjusted for multiple comparisons using Tukey test. *p-value < 0.05. **C** GSDMC2 and GSDMC3 expression is upregulated upon succinate treatment in intestines of male wild type and female wild type and *Ptk6*−/− mice. Male *Ptk6*−/− do not display upregulation of pyroptotic genes upon succinate treatment. Intestinal sections were stained with an antibody that detects both GSDMC2 and GSDMC3. Scale bar, 50 μm. **D** Analysis of Haber et al. scRNA-seq data [27] shows that *Ptk6* and *Il25* are upregulated in tuft cell population upon helminth infection (Hb). *Gsdmc1-4* are upregulated in stem, transient amplifying (TA) and enterocyte populations in a pattern like *Ptk6*. Expression of genes in specific populations are surrounded by red dashed lines.

To further understand the effect of *Ptk6* disruption on type 2 immunity, we examined the expression of genes that are upregulated following activation of the innate immune response [5, 13, 40, 41]. We focused on mediators of effector functions: enzymes for eicosanoid synthesis, cytokines, and pyroptotic genes. Several of these genes are significantly upregulated in succinate-treated wildtype and female *Ptk6−/−* mice in our dataset (Fig. 5A). We used qRT-PCR to validate RNA-seq data for cytokines *Il25* and *Il13* and observed an upward trend in the expression of these genes in wildtype males and females, as well as *Ptk6*−/− females, in response to succinate treatment (Fig. 5B).

Genes encoding GSDMCs, regulators of pyroptosis, are one of the most significant classes of DEGs in our dataset (Table S2). Succinate-treated females and wild type males exhibited strong upregulation of GSDMC2 and GSDMC3 in the jejunum, while male *Ptk6*−/− mice failed to upregulate these proteins (Fig. 5C). This pattern of protein expression mimics the RNA expression. Analysis of the Haber et al. scRNA-seq dataset [27] revealed upward trend in *Il25* and *Ptk6* expression in tuft cells upon activation of type 2 immunity with Hb (Fig. 5D). Furthermore, *Gsdmc1-4* are upregulated in the same populations as *Ptk6* (Fig. 5D).

### PTK6 regulates *Irag2* and *Il25* expression required for tuft cell mediated activation of type 2 immunity in organoids developed from male mice

**Fig. 6:**
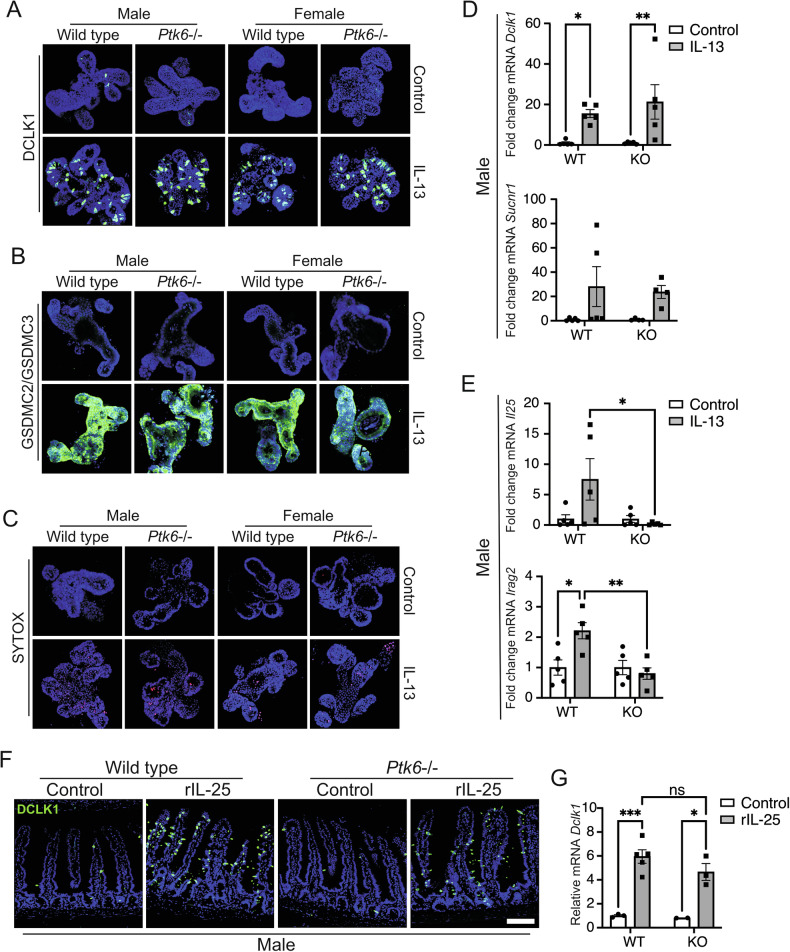
Impaired induction of *Il25* and *Irag2* in organoids developed from male *Ptk6*−/− mice following treatment with IL-13. **A** *Ptk6* disruption does not affect tuft cell differentiation. Organoids were treated for 7 days with 30 ng/ml IL-13 and visualized using anti-DCLK1 antibody. IL-13, a cytokine secreted in vivo by ILC2s upon activation of tuft cells, induced expansion of tuft cells in organoids developed from intestines of male and female wild type and *Ptk6*−/− mice. **B** Gasdermins GSDMC2 and GSDMC3 are upregulated following IL-13 treatment in organoids derived from all mice. **C** Cell death is induced with IL-13 in all organoids, visualized with SYTOX-red. Organoids were treated with SYTOX-red 30 min prior fixation and staining with DAPI. **D** Tuft cell marker genes *Dclk1* and *Sucnr1* are induced after IL-13 treatment in organoids derived from wild type and *Ptk6*−/− male mice. Gene expression is normalized against *Rps17* and shown as the fold change per genotype. **E** Upregulation of *Il25* and *Irag2* mRNA expression is impaired in organoids produced from *Ptk6*−/− male mice following IL-13 treatment. Gene expression is normalized against *Rps17* and is shown as the fold change per genotype**. F** Exogenous IL-25 rescued activation of type 2 immunity in *Ptk6*−/− male mice. Wild type and *Ptk6*−/− have similar induction of tuft cells after injection for 7 days with rIL-25. Tuft cells are visualized with anti-DCLK1 antibody. Scale bar, 50 μm. **G** Relative expression of *Dclk1* confirms upregulation of tuft cells upon rIL-25 administration to male *Ptk6*−/− mice. Gene expression is normalized against *Rps17*. For all experiments: Graphs depict mean ± SEM. Statistics: Two-way ANOVA, adjusted for multiple comparisons using Tukey test. *p-value < 0.05, **p-value < 0.01, ***p-value < 0.001.

IL-13 treatment of intestinal organoids provides effective in vitro model for studying activation of type 2 immunity [30, 36], eliminating contributions of other immune cells and the microbiome to intestinal epithelium. IL-13 is normally produced by activated ILC2s, and organoids treated with IL-13 exhibit upregulation of tuft, goblet cell, and Paneth gene signatures [30]. We treated organoids developed from jejunal epithelium, with IL-13, and observed similar induction of tuft cells in organoids from male and female wild type and *Ptk6*−/− mice (Fig. 6A). No differences in GSDMC2 and GSDMC3 expression (Fig. 6B), or cell death, visualized by staining with SYTOX-red, were observed between the sexes or genotypes (Fig. 6C). The tuft cell marker genes *Dclk1* and *Sucnr1* were induced to a similar extent in organoids derived from the male and female mice of both genotypes (Fig. 6D, S3A). These data suggest that regulation of differentiation by IL-13 is intact in male and female *Ptk6*−/− mice and indicate that the defect in *Ptk6*−/− male mice is upstream of IL-13.

Using qRT-PCR, we examined the expression of *Il25*, the tuft cell-specific cytokine that activates type 2 immunity in vivo. Interestingly, upregulation of *Il25* was impaired in organoids developed from male *Ptk6−*/− intestinal cells, showing an expression pattern like intact animals (Fig. 6E). Expression of *Irag2* is also impaired in male *Ptk6*−/− organoids (Fig. 6E). The product of *Irag2*, LRMP, mediates regulation of Ca2+ influx through interaction with IP3Rs, which is required for opening of transient receptor potential cation channel subfamily M member 5 (TRPM5) to induce membrane depolarization and IL-25 secretion [3, 42]. In organoids derived from female mice, expression of *Il25* and *Irag2* follows an upward trend after IL-13 treatment, but does not reach statistical significance (Fig. S3B). These data indicate that loss of PTK6 leads to a defect in effector functions of tuft cells in epithelial organoids developed only from males. Females may have higher basal expression of these genes, or there may be intermediate factors that more robustly activate their expression. Using scRNA-seq data from organoids [36], we confirmed that *PTK6* and *IRAG2* are expressed in the same tuft cell types upregulated upon IL-13 treatment and localized to the same cells (Fig. S3C, D).

To determine if impaired activation of innate immunity is dependent on lack of IL-25 in *Ptk6*−/− male mice, we asked if ectopic administration of IL-25 could rescue the secretory cell defects. We administered rIL-25 intraperitoneally to wild type and *Ptk6−/−* male mice. Exogenous IL-25 rescued activation of type 2 immunity in *Ptk6*−/− mice, detected by upregulation of tuft cells and *Dclk1* in jejunums from male *Ptk6*−/− mice (Fig. 6F, G). Goblet cell differentiation was also rescued in male *Ptk6−/−* mice as visualized with Alcian blue staining (Fig. S4A). Exploring the expression of two genes impacted by *Ptk6* loss, we show that *Irag2* had upward trend in wild type male mice upon IL-25 treatment, while in *Ptk6*−/− males it remained unchanged. On the other hand, *Il25* expression was rescued in male *Ptk6*−/− mice (Fig. S4B), suggesting that the administration of exogenous IL-25 can bypass the requirement for PTK6. These data further suggest that there are no immune cell defects in male *Ptk6*−/− mice, and that restoration of IL-25, expressed specifically by tuft cells, is sufficient to rescue ILC2 activation in *Ptk6−/−* male mice.

### PTK6 is expressed in tuft cells of noninflamed ileum from Crohn’s disease patients

**Fig. 7:**
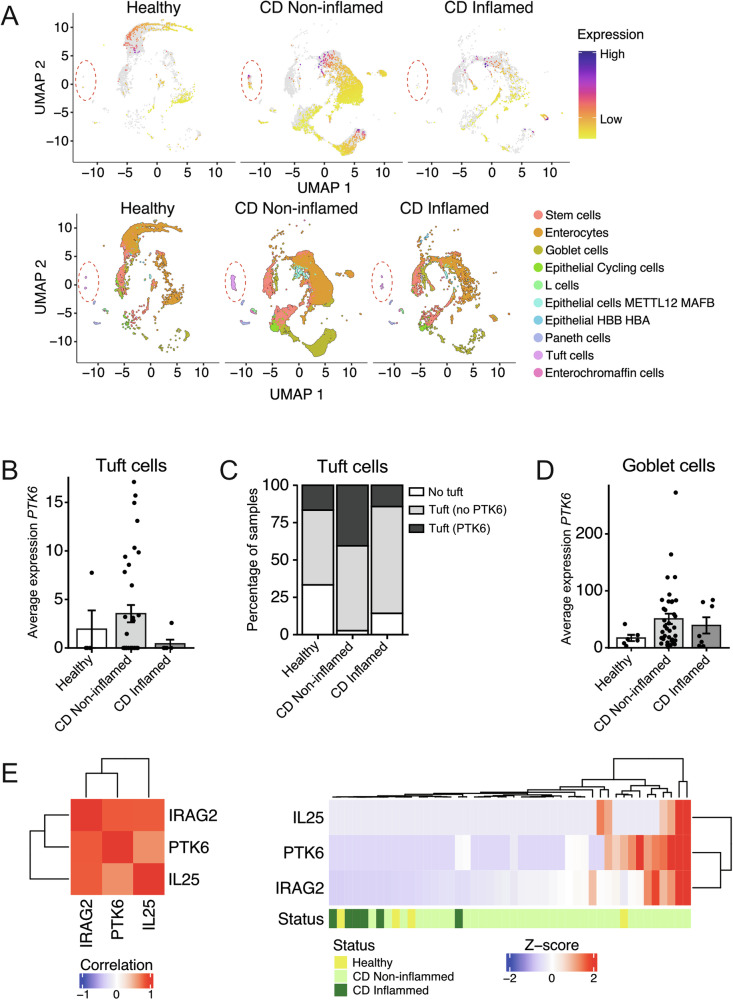
PTK6 is upregulated in tuft cells from noninflamed intestine in Crohn’s disease patients. **A** *PTK6* is highly expressed in enterocytes and goblet cells in the ileum in patients with Crohn’s disease (CD) and healthy controls. Analysis of scRNA-seq data from Kong, et al. [44] revealed expression of *PTK6* in other populations, including tuft cells (surrounded by red dashed circles). The bottom panel shows different cell populations in each sample group. All stem populations are grouped as “Stem cells”; all enterocyte populations are grouped as “Enterocytes”; all goblet cell populations are grouped as “Goblet cells”, following authors’ annotations [44]. **B** Average expression of *PTK6* in tuft cells per patient from Kong et al. scRNA-seq data [44]. **C** *PTK6* is expressed in tuft cells from a subset of samples. It is mostly expressed in tuft cells from non-inflamed CD tissues, and to a lesser extent in healthy controls and inflamed CD tissues. **D** *PTK6* is expressed in goblet cells from human patients. **E** Correlation matrix and expression heatmap of *PTK6*, *IL25* and *IRAG2* from Kong, et al. dataset [44]. Expression of *PTK6* per sample (sum of cell expression, per cell type) positively correlates with expression of *IL25* and *IRAG2* (PTK6-IRAG2: *r* = 0.81, *p*-value < 0.0001; PTK6-IL25: *r* = 0.64, *p*-value < 0.0001). Heatmap shows Z-scores of summed expression for each of the genes per cell type. *IL25* is upregulated in tuft cells in a subset of non-inflamed tissues only. It is co-expressed with *PTK6* in 5/6 samples.

Alterations in tuft cell production have been reported in patients with inflammatory bowel disease (IBD). Banerjee and colleagues showed roles for tuft cells and the activation of type 2 immunity in suppression of the IL-23 - IL-17 axis of type 3 immunity characteristic for Crohn’s disease (CD), followed by reconstitution of the epithelium [7]. Other studies report alterations in tuft cell production in CD [43, 44], as well as in ulcerative colitis (UC) [45]. To further understand how our findings might relate to human disease, we examined scRNA-seq data from ileal tissues of patients with CD or healthy controls [44]. Greater numbers of tuft cells were present in noninflamed ileal tissues of patients with CD [44] (Fig. 7A), and PTK6 was higher in these samples (Fig. 7B, C). Like findings of Banerjee and colleagues [7], inflamed intestines had reduced numbers of tuft cells [44] when compared with non-inflamed intestine (Fig. 7C). *PTK6* mRNA is expressed in tuft cells in ~15% of samples from healthy and inflamed tissues, while it is found in double the number of patients with tuft cells localized in non-inflamed regions (40%) (Fig. 7C). We did not observe significant differences in *PTK6* expression in tuft cells between sexes (Fig. S5), as already observed (Fig. 2C). Correlation analysis shows high correlation between *PTK6*, *IL25* and *IRAG2* (Fig. 7E). *IL25* was expressed only in tuft cells from noninflamed samples, and *PTK6* was co-expressed with *IL25* in 5/6 of samples (Fig. 7E), supporting potential crosstalk in tuft cells.

Using the same dataset [44], we show that *PTK6* is highly expressed in goblet cells in all groups of human samples (Fig. 7D). Together these data indicate that PTK6 may function in epithelial cells with roles in mucosal immunity, and it may regulate gene expression and/or function directly, or indirectly through regulating IL-25 expression and/or secretion.

## Discussion

Research in the last decade has revealed important differences in biology between sexes, and here we report significant differences between male and female mice dependent on PTK6 (Fig. 1). Gene set enrichment pathway analysis identified the “innate immune response in mucosa” as one of the top differences between wildtype and *Ptk6*−/− animals in both sexes (Fig. 1D) and prompted us to further explore the role of PTK6 in innate immunity. We detected upregulation of *Ptk6* upon infection with helminths and bacteria (Fig. 2A, B). Looking closely at the pathways, *Ptk6* is upregulated in tuft cells upon activation of innate type 2 immunity in vivo and in vitro (Fig. 2B, D).

We show that PTK6 regulates tuft cell-mediated activation of type 2 immunity and secretory cell expansion in the intestines of male mice. Sex differences in innate type 2 immunity in the intestine are not well understood. Activation of innate type 2 immunity in various organs shows a strong sex bias, with females having stronger and more robust immune responses [46]. Sex hormones may regulate sex-bias in innate immunity. Androgens negatively regulate ILC2s, and ILC2s express higher levels of the androgen receptor [47, 48].

Female mice produce more tuft and goblet cells than males following succinate treatment (Figs. 3A, 4A), exhibit higher expression of tuft cell markers and stronger activation of type 2 immunity (Figs. 3–5). Female wild type and *Ptk6*−/− mice exhibit some differences in gene expression (Fig. 1), but they have similar upregulation of genes involved in tuft cell-mediated type 2 immunity. Expression of FRK and SRMS, two PTK6 family members, did not significantly change in female *Ptk6−/−* mice after succinate treatment (Fig. S6A, B). *FRK* is expressed in male and female tuft cells, and in tuft cells induced by IL-13 treatment (Fig. S6C, D).

Ectopic administration of the ILC2 cytokine IL-13 induced differentiation of tuft cells in wild type and *Ptk6*−/− organoid cultures (Fig. 6A), indicating stem cell responses are intact in the absence of PTK6. While IL-13-treated wild type and *Ptk6*−/− organoids produced equivalent numbers of tuft cells, we detected reduced expression of *Il25* and *Irag2* in male *Ptk6−/−* organoids compared with wild type organoids (Fig. 6E). Defects in IL-25-dependent effector functions in vivo would lead to impaired succinate-induced activation of ILC2s, secretion of IL-13 and secretory cell differentiation. Restoring IL-25 by administration of rIL-25 to *Ptk6*−/− male mice stimulated tuft cell differentiation to wild type levels (Fig. 6F).

Regulation of IL-25 in tuft cells at transcriptional, translational, and post-translational levels is not well understood. Epigenetic mechanisms may be involved, since sex-differences in PTK6 regulated *Il25* expression are maintained in organoid cultures. Rescue of *Il25* expression following administration of exogenous IL-25 (Fig. S4B) suggests that IL-25 may auto-regulate itself [49–51] independently of PTK6, and we cannot rule out induction of IL-25 transcription in rIL-25 treated mice by PTK6-independent mechanisms. IL-25 has been shown to be part of an autoregulatory loop that activates STAT3 [50, 51]. Interestingly, PTK6 regulates activation of STAT3 through direct tyrosine phosphorylation [25, 52], and STAT3 activation was impaired in *Ptk6*−/− male mice [25]. It is possible that impaired STAT3 activation could be corrected through activation of STAT3 by rIL25.

In humans, the *IL25* gene maps to an inflammatory bowel susceptibility locus (14q11-12, IBD4). However, no mutations or polymorphisms were identified in the *IL25* gene, leading investigators to conclude it had no association with IBD [53]. However, they did not consider contributions of transcriptional and posttranscriptional regulation. IL-25-mediated activation of type 2 immunity may have beneficial effects for patients with CD. Banerjee and colleagues reported a reduced number of tuft cells in inflamed ileums from patients and in a mouse model of CD [7]. PTK6 is expressed in tuft cells in almost half of patients in non-inflamed CD (Fig. 7C), and its expression shows an upward trend in tuft cells from noninflamed regions of CD ileums (Fig. 7B). Co-expression of *IL25* and *IRAG2* with *PTK6* in a subset of patients with noninflammatory CD further suggests crosstalk and the potential for PTK6 to regulate tuft cells and inflammation in CD.

Understanding contributions of sex to disease is critical for precision medicine. For example, sex differences impact the prevalence, risk, and outcomes of autoimmune and cardiovascular diseases and cancer [54]. In the intestine, type 2 immune responses are important for clearing certain infections, such as helminths, which can lead to malnutrition, vitamin deficiencies and intestinal obstructions [55]. Resistance to existing anthelmintics is a public health concern and understanding mechanisms of helminth clearance will be important for developing novel treatments [56]. Understanding the sex-biased functions of PTK6 in regulation of innate immunity may lead to improved strategies for treatment of a variety of sex-related diseases.

## Methods and materials

### Mice

Animal experiments were approved by the University of Illinois at Chicago Institutional Animal Care and Use Committee. All mice were maintained under specific pathogen-free conditions. The generation of the germline knockout of *Ptk6*^*−/−*^ has been described [18]. This strain has been backcrossed more than 20 generations into the C57BL/6 background and has been made available at The Jackson Laboratory Repository with the JAX Stock No. 038346. All experiments utilized aged-matched animals from different litters, from the same breeding colony. All experiments ended at 9–11 weeks of age. For experiments on activation of type 2 immunity, male and female wild type and *Ptk6*−/− mice were given 100 mM succinate (S9637; Sigma Aldrich, St. Louis, MO) in drinking water for 7 days [11]. For rescue experiments with IL-25, male wildtype and *Ptk6*^*−/−*^ mice were injected intraperitoneally 0.5 μg of rIL-25 (1399-IL; R&D systems, Minneapolis, MN) diluted in PBS, or PBS only for controls. Tissues were harvested and paraffin-embedded for histology or processed for RNA isolation. Jejunums were divided for immunohistochemistry (proximal and middle) and RNA preparation (distal).

### Organoid cultures

Organoids were generated from small intestines of 5 male wild type, 5 female wild type, 5 male *Ptk6*−/− and 5 female *Ptk6*−/− mice according to Sato et al. [57] following the media manufacturer’s protocol (STEMCell Technologies, Vancouver, Canada). Briefly, proximal jejunums from male and female 8–9 weeks old mice were harvested, 8 cm of the proximal small intestine was washed, villi scraped off, and the intestine was cut into 2 mm pieces. The pieces were washed with PBS 10 times and incubated with 3 mM EDTA in PBS for 30 min at 4 °C with gentle rocking. The crypts removal was facilitated by vigorous shaking, and crypts were passed through a 70 μm cell strainer (352350; Corning, Corning, New York). Crypts were washed in DMEM/F-12, counted, and resuspended in Matrigel (356231; Corning, Corning, New York) in a ratio of 2:3 (60 μl) with a final count ~700 crypts/well. After establishing the culture, organoids were passaged every 6–7 days using Gentle Cell Dissociation Reagent (100–0485, STEMCell Technologies). Experiments were performed between the 2^nd^ and 4^th^ passage.

To mimic the activation of type 2 immunity and promote tuft cell differentiation in culture, organoids were treated with 30 ng/ml IL-13 and/or 10 mM sodium-succinate, and at days 1, 3, and 5 after plating. Organoids were plated in 24-well plates for RNA analysis (6 wells per treatment), or ibidi 8-well chambers with glass bottoms (80827; Ibidi, Fitchburg, WI) for immunofluorescence (at least 2 wells per treatment per antibody). The addition of sodium-succinate alone or in combination with IL-13 had no impact on tuft cell differentiation in the organoid models.

### Pyroptosis analysis

Organoids were plated in each well of ibidi 8-well chambers, in 30 μl of 2:3 ratio of organoids/Matrigel mix per well. SYTOX-red (S34859; Invitrogen, Waltham, MA) was added to the medium at the concertation 0.5 μM, 30 min before the end of the experiment. After treatment, the organoids were washed, fixed in 4% paraformaldehyde for 30 min, and further analyzed.

### Immunohistochemistry and immunofluorescence

Tissues containing proximal/middle jejunums were rehydrated and antigen retrieval was performed using sodium-citrate buffer pH 6, at 95 °C for 20 min. For DAB staining, tissues were incubated with H_2_O_2_ for 10 min. Tissues were blocked in 5% BSA supplemented with 2% of appropriate serum based on the source of the secondary antibody, for 1 h at room temperature. Primary antibodies were incubated overnight at 4 °C. Secondary antibodies were incubated for 1 h following the appropriate detection. DAB-stained tissues were counterstained with Hematoxylin dye, while immunofluorescent tissues were counterstained with 4′,6-diamidino-2-phenylindole (DAPI; Invitrogen, Waltham, MA). The number of tuft cells is quantified as the number of DCLK1+ cells per crypt-villus. For the quantification, 4–5 of random areas from each tissue were taken with Leica DM8 fluorescent microscope at 10x magnification.

For organoid experiments, fixed organoids were permeabilized using 0.2% Triton-X in PBS for 30 min, washed in 0.1% Tween in PBS (PBST), and blocked in 5% BSA in PBST overnight at 4 °C. Primary antibodies were incubated overnight at 4 °C, secondary antibodies for 1.5 h at room temperature, and streptavidin-Alexa Fluor for 30 min at room temperature. Nuclei were stained with DAPI.

### Antibodies

Primary antibodies used in the study: anti-DCLK dilution 1:2000 (ab31704; Abcam, Cambridge, UK), anti-GSDMC2/GSDMC3 dilution 1:2500 (Abcam), anti-Lysozyme dilution 1:200 (A0099, DakoCytoman, Denmark), anti-Ki-67 1:200 (ab16667, Abcam).

### Alcian blue staining of goblet cells

Deparaffinized and rehydrated tissues were incubated in 3% glacial acetic acid solution prior to incubation with 1% w/v Alcian Blue solution (A3157; Sigma Aldrich, St. Louis, MO). Tissues were counterstained with Nuclear Fast Red (H3403; Vector Laboratories, Newark, California). The number of goblet cells is quantified as the number of Alcian Blue+ cells per crypt-villus. For quantification of goblet cells, 4–5 of random areas from each tissue were taken with Leica DM8 light microscope at 10x magnification.

### Quantitative RT–PCR

Total RNA was isolated from distal jejunums using TRIzol (15596026, Invitrogen), following the manufacturer’s protocol. cDNA was prepared from total RNA using iScript (1708891; Bio-Rad, Hercules, CA), and qPCR was performed using SensiFAST SYBR No-ROX Kit (BIO-98005; Meridian Life sciences, Cincinnati, OH) following the manufacturer’s protocols. Primers used in the study:

*Dclk1*: Fwd: 5′-CAGCCTGGACGAGCTGGTGG-3′, and Rev: 5′-TGACCAGTTGGGGTTCACAT-3′; *Il13*: Fwd: 5′-TGAGCAACATCACACAAGACC-3′, and Rev: 5′-GGCCTTGCGGTTACAGAGG-3′; *Il25*: Fwd: 5′-ACAGGGACTTGAATCGGGTC-3′, and Rev: 5′-TGGTAAAGTGGGACGGAGTTG-3′; *Irag2*: Fwd: 5′-TTTGGACGTGACAAGAGGGT-3′, and Rev: 5′-ACTCTGGTCCTCGTTCATTCTG-3′; *Muc2*: Fwd: 5′-ATGCCCACCTCCTCAAAGAC-3′, and Rev: 5′-GTAGTTTCCGTTGGAACAGTGAA-3′; *Rps17*: Fwd: 5′-CGCCATTATCCCCAGCAAG-3′, and Rev: 5′-TGTCGGGATCCACCTCAATG-3′; *Sucnr1*: Fwd: 5′-GGCAGAGTTTTCTGTCGAGAC-3′, and Rev: 5′-ACATTCCCAAGCAGTCCAA-3′.

For all experiments, gene expression was normalized to *Rps17* using the 2^-ΔΔCT^ method. When analyzing gene expression in tissues, expression is shown relative to control wild type mice, for which average expression is set at 1. For organoids, gene expression is shown as the fold change in gene expression following each treatment, averaged per mouse genotype.

### Bulk RNA-seq

Total jejunal RNA samples were processed by LC Sciences (Houston, TX). RNA quantity and purity were analyzed using Bioanalyzer 2100 and RNA 6000 Nano LabChip Kit (Agilent, CA, USA, 5067-1511), and high-quality RNA samples with RIN number > 7.0 were used to construct a sequencing library. mRNA was purified from total RNA (5ug) using Dynabeads Oligo (dT) (Thermo Fisher, CA, USA) with two rounds of purification, and fragmented into short fragments using divalent cations under elevated temperature (Magnesium RNA Fragmentation Module (NEB, cat. e6150, USA) under 94 °C 5–7 min). cDNA was generated using SuperScriptTM II Reverse Transcriptase (Invitrogen, cat. 1896649, USA). cDNA was then used to synthesize U-labeled second-stranded DNAs with E. coli DNA polymerase I (NEB, cat.m0209, USA), RNase H (NEB, cat.m0297, USA) and dUTP Solution (Thermo Fisher, cat. R0133, USA). An A-base was added to the blunt ends of each strand, preparing them for ligation to the indexed adapters. Each adapter contained a T-base overhang for ligating the adapter to the A-tailed fragmented DNA. Dual-index adapters were ligated to the fragments, and size selection was performed with AMPureXP beads. After the heat-labile UDG enzyme (NEB, cat.m0280, USA) treatment of the U-labeled second-stranded DNAs, the ligated products were amplified with PCR by the following conditions: initial denaturation at 95 °C for 3 min; 8 cycles of denaturation at 98 °C for 15 sec, annealing at 60 °C for 15 sec, and extension at 72 °C for 30 sec; and then final extension at 72 °C for 5 min. The average insert size for the final cDNA libraries was 300 ± 50 bp. At last, we performed the 2×150 bp paired-end sequencing (PE150) on an Illumina NovaseqTM 6000 following the vendor’s recommended protocol.

### RNA-seq bioinformatics analysis

Using Illumina paired-end RNA-seq approach, we sequenced the transcriptome, generating a total of million 2 ×150 bp paired-end reads. Reads were further filtered by Cutadapt (https://cutadapt.readthedocs.io/en/stable/,version:cutadapt-1.9). The parameters were as follows: 1) removing reads containing adapters; 2) removing reads containing polyA and polyG; 3) removing reads containing more than 5% of unknown nucleotides (N); 4) removing low-quality reads containing more than 20% of low quality (Q-value ≤ 20) bases. Sequence quality was verified using FastQC (http://www.bioinformatics.babraham.ac.uk/projects/fastqc/, 0.11.9). including the Q20, Q30, and GC content of the clean data. Reads were aligned with the reference genome using the HISAT2 package (https://daehwankimlab.github.io/hisat2/,version:hisat2-2.0.4). The mapped reads of each sample were assembled using StringTie (http://ccb.jhu.edu/software/stringtie/,version:stringtie-1.3.4d) with default parameters. Then, all transcriptomes from all samples were merged to reconstruct a comprehensive transcriptome using gffcompare software (http://ccb.jhu.edu/software/stringtie/gffcompare.shtml,version:gffcompare-0.9.8). After the final transcriptome was generated, StringTie and ballgown (http://www.bioconductor.org/packages/release/bioc/html/ballgown.html) were used to estimate the expression levels of all transcripts and perform expression abundance for mRNAs by calculating FPKM (fragment per kilobase of transcript per million mapped reads) value.

Differential gene expression analysis was performed using DESeq2 software [58] between two different groups (and by edgeR [59] between two samples). The genes with the parameter of false discovery rate (FDR) below 0.05 and absolute fold change ≥ 2 were considered differentially expressed genes.

Gene set enrichment analysis was performed using software GSEA (v4.1.0) and MSigDB to identify whether a set of genes in specific GO terms, KEGG pathways, DO terms, and Reactome shows significant difference between groups [60]. Briefly, we input gene expression matrix and rank genes by Signal2Noise normalization method. Enrichment scores and *p*-value was calculated in default parameters. GO terms, KEGG pathways (DO terms, Reactome) meeting this condition with |NES| > 1, NOM p-val<0.05, FDR *q*-val < 0.25 were considered to be different in two groups.

Whole raw and processed data files have been deposited in NCBI’s Gene Expression Omnibus [61] and are accessible through GEO Series accession number GSE243229. Other RNA-seq and scRNA-seq are publicly available from the referenced studies [27, 28, 44]

### Public data analysis and statistics

RNA-seq data were explored for significant gene expression, and compared with previously identified marker genes of tuft cells [27], and type 2 immunity-related pyroptotic genes [13].

Publicly available RNA-seq data was obtained directly from El-Naccache et al. [28], and expression normalized to control values (naïve mice).

Publicly available single cell (sc) RNA-seq data were obtained from GEO (Gene Expression Omnibus, https://www.ncbi.nlm.nih.gov/geo/): GSE92332 [27] and GSE233451 [36]; from CZ CELL×GENE Discover [62]: Gut cell atlas [35]; from Broad Institute Single Cell Portal: SCP1884 [44]. Tuft cells from Haber et al. [27] originate from mouse small intestine, Kong et al. [44] from human ileum, while tuft cells from other studies [35, 36] were not separated based on tissue origin. For Elmentaite et al. study [35] we used only expression from healthy samples. The pseudo-bulk analysis was performed from data analyzed following authors’ preprocessing steps and annotated cell types based on the author’s information for all samples together or already available normalized data grouped per sample. Statistical analysis of *Ptk6* upregulation upon infection [27] was performed using monocle3 [63–65]. Other single-cell analyses were performed based on the authors’ guidance and available markers for cell type annotations: Elmentaite et al. [35], and Kong et al. [44]: cell annotations originated from metadata, expression values from normalized matrix and visualized using with monocle3 [63–65] without normalization; Haber et al. [27]: cell annotations originated from metadata, SalmHelm UMI counts matrix was normalized using monocle3 default settings. Huang et al. [36]: gene expression normalized by following authors’ guidelines, cell populations grouped based on metadata.

Data were analyzed using R software. For the analysis of scRNA-seq data, we used the Seurat package (version 5) [66] and monocle3 [63–65]. For visualization, we used scCustomize [67], ggplot2 [68] and ComplexHeatmap packages [69].

Statistical analyses for quantification and qPCR data were performed using GraphPad Prism software version 9 (La Jolla, CA); two-way ANOVA and Mann-Whitney tests were used to determine significant differences between groups. *p-value < 0.05, **^,^^##^*p*-value < 0.01, ***^,^^###^*p*-value < 0.001, ****p-value < 0.0001. # is used for comparison within the same sex when shown on the same graph with all comparisons.

## Supplementary information

Supplemental Text and Figures

Supplemental Table 1

Supplemental Table 2

## Data Availability

Processed RNA-seq data from jejunums of wild type and *Ptk6*−/− mice are available in the article and supplementary materials. Additionally, the whole raw and processed data files have been deposited in NCBI’s Gene Expression Omnibus [61] and are accessible through GEO Series accession number GSE243229. Other datasets used in this study are listed in sections “Methods and Materials” and “Supplemental Material and Methods.” This study did not generate any new code.
