# Supplementary material for: Impaired activation of succinate-induced type 2 immunity and secretory cell production in the small intestines of Ptk6−/− male mice
Source: Cell Death Dis. 2024 Oct 26;15(10):777. doi: 10.1038/s41419-024-07149-9 (PMC11513114; doi:10.1038/s41419-024-07149-9)
Supplement: Supplementary file 1 — Supplemental Text and Figures [file 41419_2024_7149_MOESM1_ESM.pdf]

SUPPLEMENTARY INFORMATION FOR:

Impaired activation of succinate-induced type 2 immunity and secretory cell production in the small intestines of *Ptk6-/-* male mice

Katarina Vlajic, Wenjun Bie, Milica B. Gilic, and Angela L. Tyner

1. Supplementary figure legends
2. Supplementary table legends

SUPPLEMENTARY FIGURE LEGENDS

Figure S1. Analysis of gene expression and cell types related to activation of type 2 immunity. A) and **B)** Heatmaps of significantly expressed genes in our RNA-seq data of markers for type-2 and type-1 tuft cells as identified by Haber and colleagues (1). Succinate treatment primarily upregulates type-2 tuft cell marker genes. **C)** PTK6 expression in goblet cells of human gastrointestinal tract (2). Plots show changes based on developmental stage or sex.

Figure S2. Proliferation is unchanged following succinate treatment of wild type and *Ptk6-/-* mice. Sections of jejunum are stained for expression of the proliferation marker Ki67. Proliferation of cells in the crypts is similar in males and females following succinate treatment of wild type and *Ptk6*-/- mice. Scale bar, 50 μm.

Figure S3. Characterization of gene expression following activation of type 2 immunity in organoids using IL-13. A) qRT-PCR was used to examine expression of tuft cell markers *Dclk1* and *Sucnr1*, in female organoids, and shows comparable induction of tuft cell marker expression upon IL-13 treatment. **B)** An upward trend in *Il25* and *Irag2* expression is detected in wild type and *Ptk6*-/- female organoids using qRT-PCR. Expression is shown as the fold change per genotype. Gene expression is normalized against *Rps17*. Expression is shown as the fold change per genotype. Graphs depict mean ± SEM. Statistics: Two-way ANOVA, adjusted for multiple comparisons using Tukey test. **p-value < 0.01, ***p-value < 0.001, ****p-value < 0.0001. **C)** and **D)** *PTK6* and

IRAG2 expression in tuft cells in organoids from Huang et al. (3). **C)** Violin plots showing *PTK6* and *IRAG2* expression in same tuft cell populations. **D)** UMAP showing overlap of *PTK6* and *IRAG2* expression in the same cells. There is no *IL25* expression in the dataset.

Figure S4. Recombinant IL-25 rescues secretory cell defects. A) Expansion of goblet cells is rescued in male *Ptk6*-/- mice upon administration of rIL-25. Representative images of Alcian Blue stained small intestines from male wild type and *Ptk6*-/- mice. Scale bar, 50 μm. **B)** Levels of *Irag2* and *Il25* expression in male mice following administration of rIL-25 suggests that regulation of *Il25* can be bypassed by exogenous rIL-25 administration. Gene expression is normalized against *Rps17*. Expression is shown as the fold change per genotype. Graphs depict mean ± SEM. Statistics: Two-way ANOVA, adjusted for multiple comparisons using Tukey test. *p-value < 0.05.

Figure S5. Detailed *PTK6* expression in patient samples. *PTK6* expression in human tuft cells is similar between sexes. The average expression of PTK6 in tuft cells in a single cell data set of Kong and colleagues from patients with Crohn's disease (4), grouped by sex and inflammation status.

Figure S6. Expression of PTK6 and PTK6 family members. A) and **B)** Expression of *Ptk6*, *Frk* and *Srms* in our RNA-seq data. **A)** Heatmap, showing z-score. **B)** Plots showing expression in fragments per kilobase of transcript per million mapped reads (FPKM). Statistical analysis for individual conditions, using t-test: Male control: *Ptk6* vs *Frk* p.adj <

0.0001; *Ptk6* in female wild type: control vs succinate p.adj = 0.0507. **C)** and **D)** FRK expression in tuft cells from scRNA-seq data (2, 3).

SUPPLEMENTARY TABLE LEGENDS

Table S1. RNA-seq data from control animals. Data show significant DEGs in males and females (FDR < 0.05, absolute fold change (fc) ≥ 2), and significant GSEA gene ontology analysis (FDR < 0.05).

Table S2. RNA-seq data from succinate-treated animals. Data is shown for a subset of genes in the dataset.

REFERENCES

1. Haber AL, Biton M, Rogel N, Herbst RH, Shekhar K, Smillie C, et al. A single-cell survey of the small intestinal epithelium. Nature. 2017;551(7680):333-9.
2. Elmentaite R, Kumasaka N, Roberts K, Fleming A, Dann E, King HW, et al. Cells of the human intestinal tract mapped across space and time. Nature. 2021;597(7875):250-5.
3. Huang L, Bernink JH, Giladi A, Krueger D, van Son GJF, Geurts MH, et al. Tuft cells act as regenerative stem cells in the human intestine. bioRxiv. 2024:2024.03.17.585165.
4. Kong L, Pokatayev V, Lefkovith A, Carter GT, Creasey EA, Krishna C, et al. The landscape of immune dysregulation in Crohn's disease revealed through single-cell transcriptomic profiling in the ileum and colon. Immunity. 2023;56(2):444-58.e5.

Figure S1

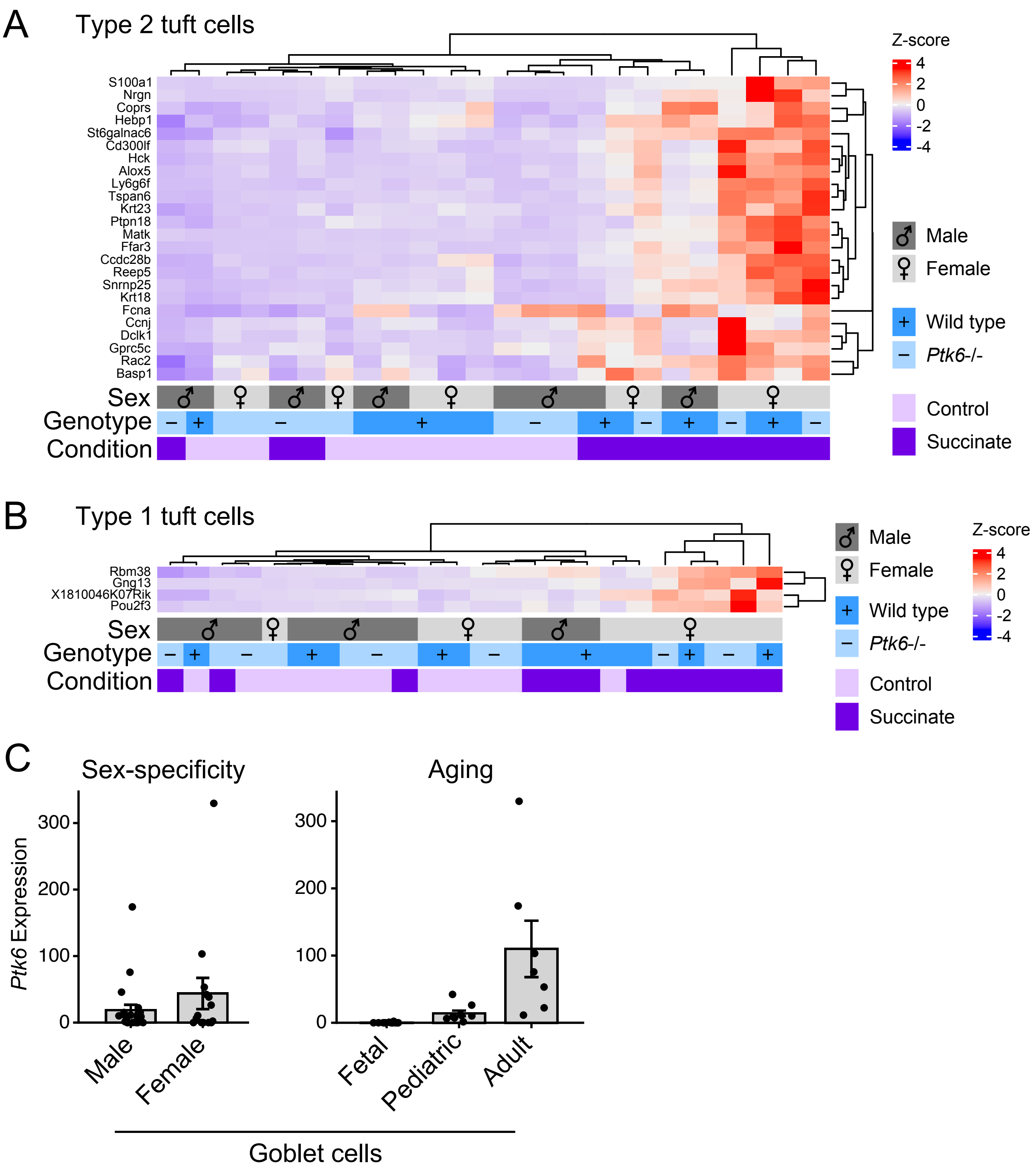
A
Type 2 tuft cells
S100a1
Nrgn
Coprs
Hebp1
St6galnac6
Cd300lf
Hck
Alox5
Ly6g6f
Tspan6
Krt23
Ptpn18
Matk
Ffar3
Ccdc28b
Reep5
Snrnp25
Krt18
Fcna
Ccnj
Dclk1
Gprc5c
Rac2
Basp1
Sex
Genotype
Condition
Z-score
4
2
0
-2
-4
Male
Female
Wild type
Ptk6-/-
Control
Succinate
B
Type 1 tuft cells
Rbm38
Gng13
X1810046K07Rik
Pou2f3
Sex
Genotype
Condition
Male
Female
Wild type
Ptk6-/-
Control
Succinate
Z-score
4
2
0
-2
-4
C
Sex-specificity
Aging
Ptk6 Expression
300
200
100
0
Male
Female
Fetal
Pediatric
Adult
Goblet cells

Figure S2

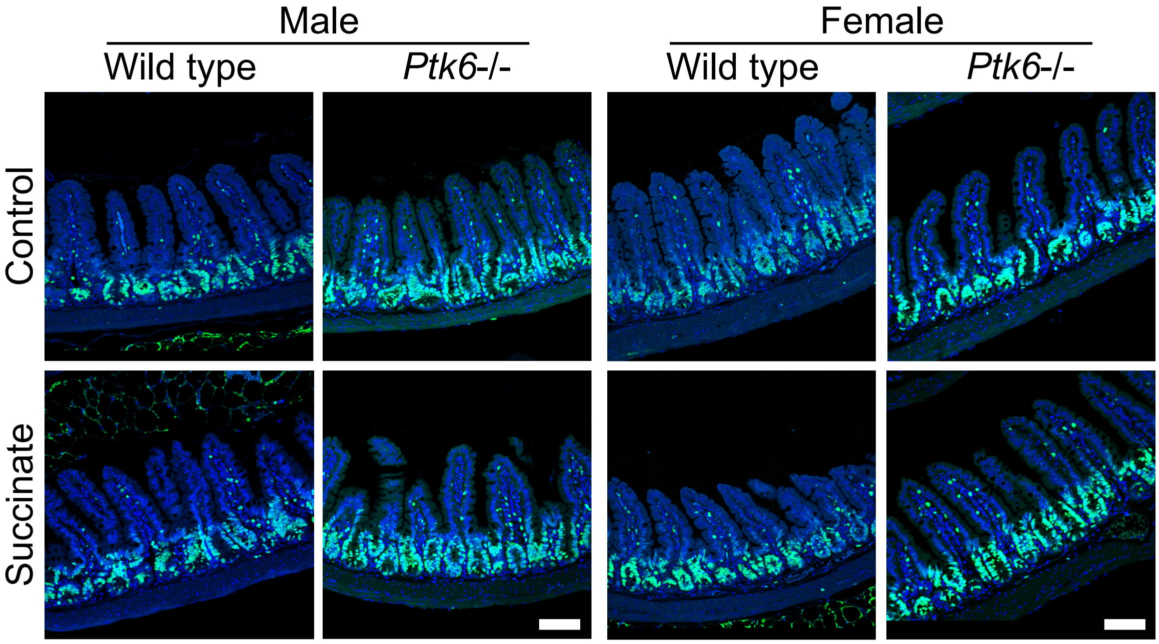
Male
Female
Wild type
Ptk6-/-
Wild type
Ptk6-/-
Control
Succinate

Figure S3

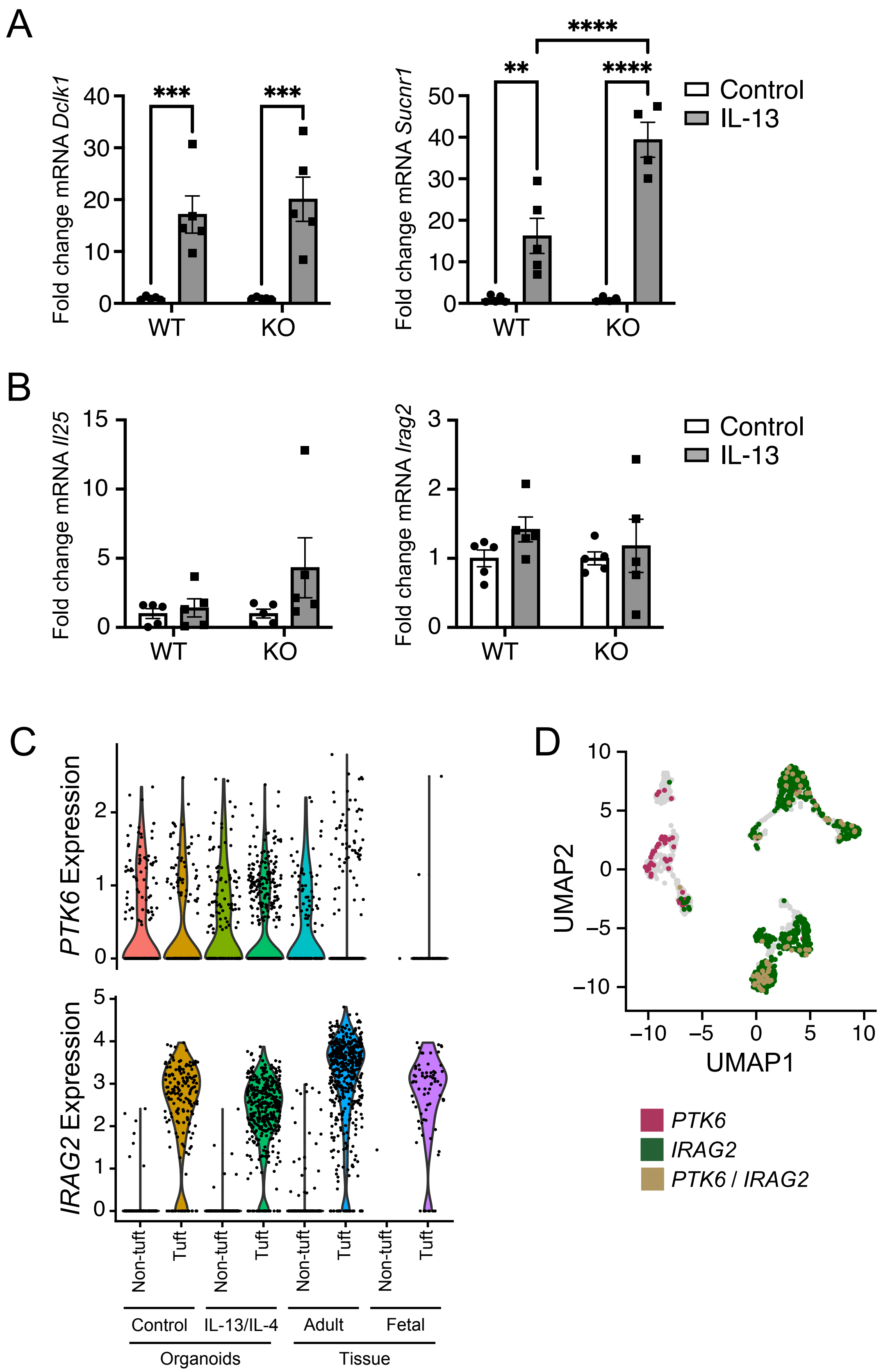
A
Fold change mRNA Dclk1
40
30
20
10
0
***
***
WT
KO
Fold change mRNA Sucnr1
50
40
30
20
10
0
**
****
****
WT
KO
Control
IL-13
B
Fold change mRNA Il25
15
10
5
0
WT
KO
Fold change mRNA Irag2
3
2
1
0
WT
KO
Control
IL-13
C
PTK6 Expression
2
1
0
IRAG2 Expression
5
4
3
2
1
0
Non-tuft
Tuft
Non-tuft
Tuft
Non-tuft
Tuft
Non-tuft
Tuft
Control
IL-13/IL-4
Adult
Fetal
Organoids
Tissue
D
UMAP2
10
5
0
−5
−10
−10
−5
0
5
10
UMAP1
PTK6
IRAG2
PTK6 / IRAG2

Figure S4

A

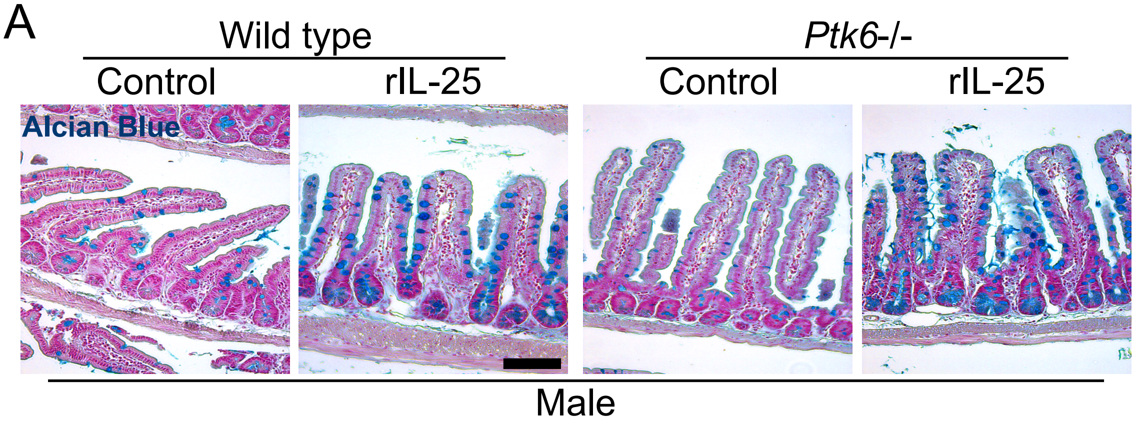

Wild type
Control
rIL-25
Ptk6-/-
Control
rIL-25
Alcian Blue
Male

B

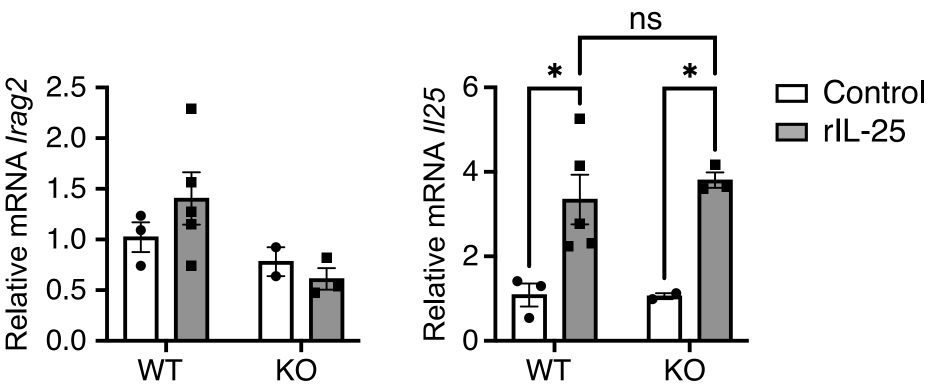

Relative mRNA Irag2
2.5
2.0
1.5
1.0
0.5
0.0
WT
KO
Relative mRNA Il25
6
4
2
0
ns
*
*
WT
KO
Control
rIL-25

Figure S5

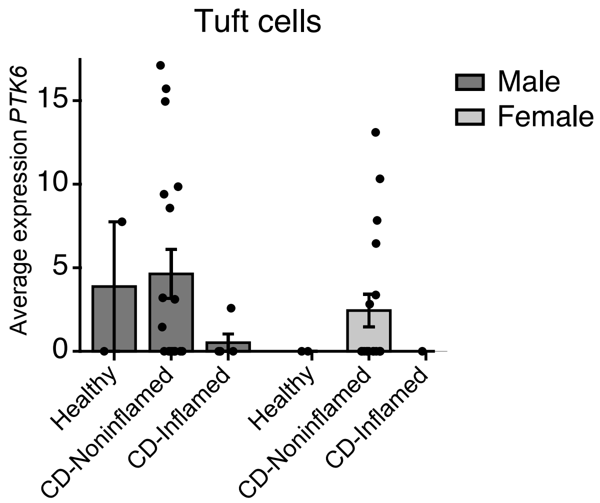

Tuft cells
Average expression PTK6
15
10
5
0
Male
Female
Healthy
CD-Noninflamed
CD-Inflamed
Healthy
CD-Noninflamed
CD-Inflamed

Figure S6

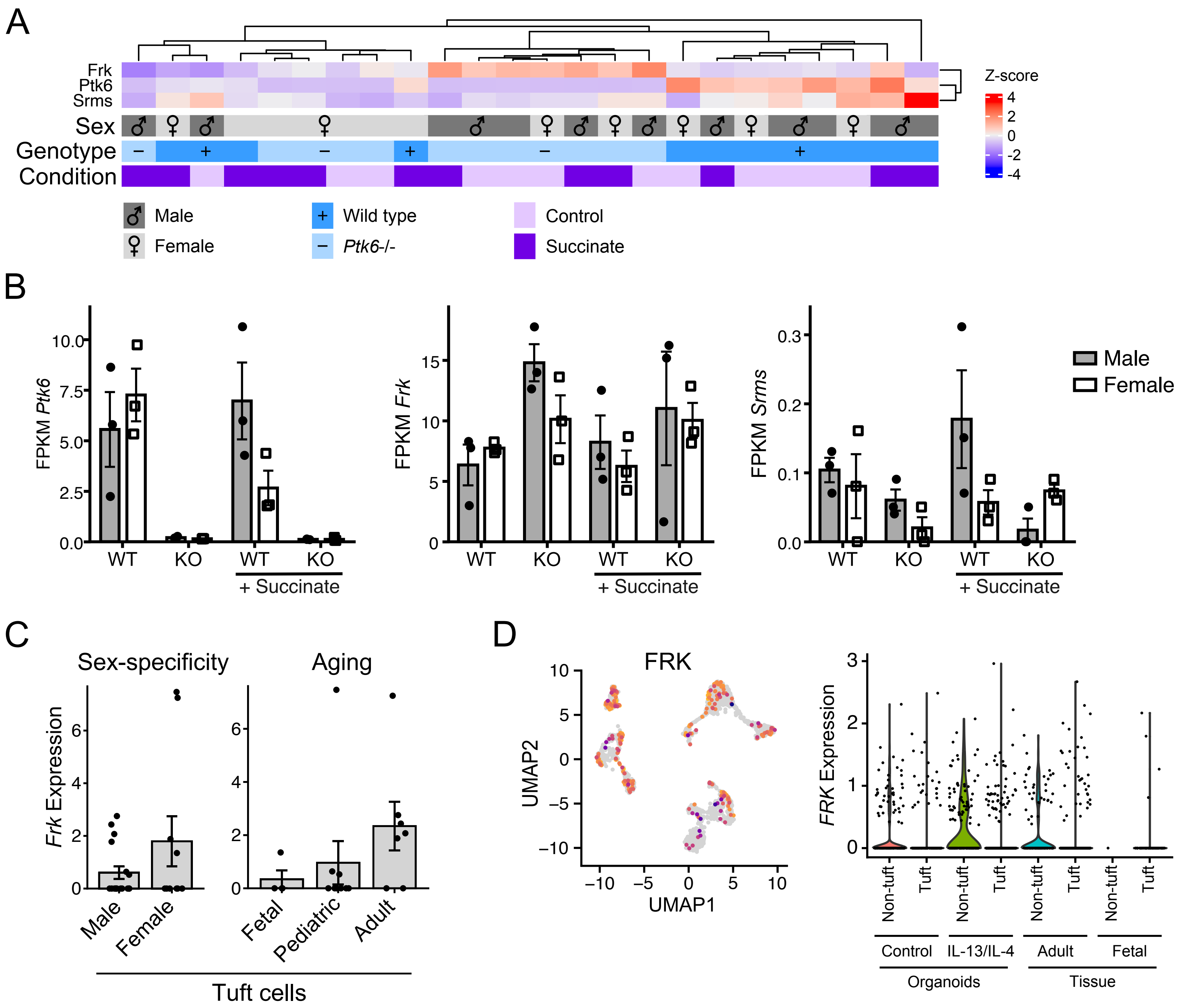
A
Frk
Ptk6
Srms
Sex
Genotype
Condition
Z-score
4
2
0
-2
-4
Male
Female
Wild type
Ptk6-/-
Control
Succinate
B
FPKM Ptk6
FPKM Frk
FPKM Srms
WT
KO
WT
KO
+ Succinate
Male
Female
C
Sex-specificity
Aging
Frk Expression
Male
Female
Fetal
Pediatric
Adult
Tuft cells
D
FRK
UMAP1
UMAP2
FRK Expression
Non-tuft
Tuft
Control
IL-13/IL-4
Adult
Fetal
Organoids
Tissue
